# Global Surveillance of *trans*-Fatty Acids

**DOI:** 10.5888/pcd16.190121

**Published:** 2019-10-31

**Authors:** Chaoyang Li, Laura K. Cobb, Hubert W. Vesper, Samira Asma

**Affiliations:** 1 Division of Global Health Protection, Center for Global Health, Centers for Disease Control and Prevention, Atlanta, Georgia; 2 Resolve to Save Lives, Vital Strategies, New York, New York; 3 Division of Laboratory Sciences, National Center for Environmental Health, Centers for Disease Control and Prevention, Atlanta, Georgia; 4 World Health Organization, Geneva, Switzerland

## Abstract

*Trans*-fatty acid (TFA) intake can increase the risk of coronary heart disease (CHD) morbidity and mortality and all-cause mortality. Industrially produced TFAs and ruminant TFAs are the major sources in foods. TFA intake and TFA-attributed CHD mortality vary widely worldwide. Excessive TFA intake is a health threat in high-income countries; however, it is also a threat in low- and middle-income countries (LMICs). Data on TFA intake are scarce in many LMICs and an urgent need exists to monitor TFAs globally. We reviewed global TFA intake and TFA-attributed CHD mortality and current progress toward policy or regulation on elimination of industrially produced TFAs in foods worldwide. Human biological tissues can be used as biomarkers of TFAs because they reflect actual intake from various foods. Measuring blood TFA levels is a direct and reliable method to quantify TFA intake.

**Summary**

**What is already known on this topic?**

*Trans*-fatty acid (TFA) intake increases the risk of morbidity and mortality due to coronary heart disease and all-cause mortality. Many low- and middle-income countries lack accurate and reliable data on TFA intake in their populations.

**What is added by this report?**

We found wide variations in global burden of TFA intake. TFA intake and TFA-attributed cardiovascular disease has decreased in high-income countries. An urgent need exists to measure and monitor TFA intake globally. Human biological tissues can be used as biomarkers of TFAs as they reflect actual intake from various foods.

**What are the implications for public health practice?**

Laboratory testing of TFA levels in plasma or serum is a direct and reliable method for estimating short-term TFA intake from foods. Measuring and monitoring TFA intake in the population is key for making evidence-based policies and evaluating the effectiveness of public health interventions.

## Background

The World Health Organization (WHO) calls for elimination of industrially produced (artificial) *trans*-fatty acids (TFAs) from the global food supply by 2023; in May 2018 WHO launched the REPLACE (review, promote, legislate, assess, create, enforce) action package to provide strategic guidance for all countries to take action toward this goal (1). One of the 6 components of the REPLACE package is to “assess and monitor trans-fats content in the food supply and changes in trans-fat consumption in the population.” Measuring the TFA levels in human biologic tissues can be an objective and reliable method for quantifying total TFA intake from foods (2–4). TFAs are derived from 2 major sources: industrially produced TFAs (iTFAs) and ruminant TFAs (rTFAs). Partially hydrogenated oils are the primary sources of iTFAs and are found in margarines, shortenings, baked goods, some popular processed and frozen foods (microwave popcorn, frozen pizza, snack foods), and fried foods (5). Dairy products and meats of ruminant animals (eg, cattle, sheep, goats) may contain small quantities of rTFAs. Both iTFAs and rTFAs consist of the same positional *trans* isomers, but they differ in distribution and amount. The number of calories in iTFAs as a proportion of the number of calories in all TFAs in the daily diet varies across countries; this proportion is estimated to range from 20% to 80% (5). In practice, because it is difficult to distinguish between iTFAs and rTFAs in human biologic tissues, total TFAs are quantified.

**Figure 1:**
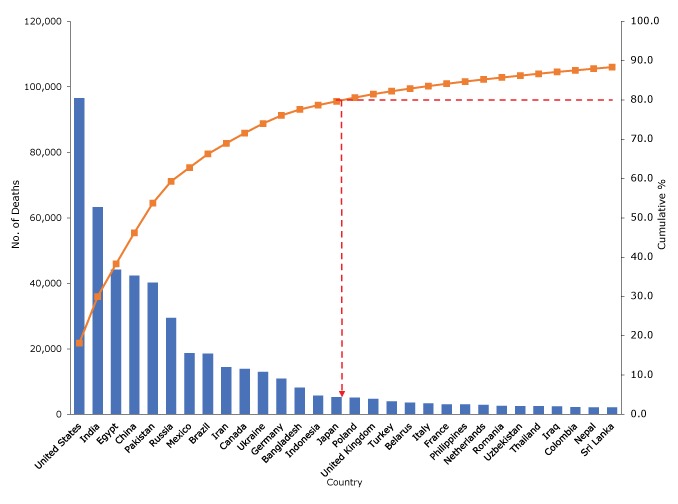
Pareto analysis on the estimated number of deaths from coronary heart disease attributable to high intake of *trans*-fatty acids (TFAs) (defined by Wang et al [8] as >0.5% of total energy intake), top 30 countries. Pareto charts are used to describe the countries in descending order of the total estimated number of CHD deaths worldwide (9). In this chart, bars indicate the estimated number of CHD deaths attributable to high TFA intake, the curved line indicates cumulative percentages, and the dashed line indicates the 15 countries that account for 80% of total CHD deaths attributable to high TFA intake worldwide according to the Pareto principle (the 80/20 rule). Data source: Wang et al (8).

Evidence from experimental studies, dietary trials, and prospective observational studies in which TFAs are measured by biomarkers or dietary records consistently shows that intake of TFAs, from either industrial or natural sources, can increase low-density lipoprotein (“bad”) cholesterol levels, decrease high-density lipoprotein (“good”) cholesterol levels, and is associated with an increased risk of coronary heart disease (CHD) morbidity and mortality and all-cause mortality (6,7). Daily intake of total TFAs varies worldwide; the estimated contribution to total energy intake ranges from 0.2% in Barbados to 6.5% in Egypt. Even small amounts of TFAs can contribute to increased risk of CHD (8). Every year, more than a half-million deaths from CHD worldwide may be attributable to high intake of TFAs (defined by Wang et al [8] as >0.5% of total energy intake); most of these deaths occur in low- and middle-income countries (LMICs) (8). Based on data published by the Global Burden of Diseases Nutrition and Chronic Diseases Expert Group (8) and application of the Pareto principle (9), 15 countries account for approximately 80% of the total number of CHD deaths attributable to high intake of TFAs globally (Figure 1). 

**Table:** 

Country	Annual No. of CHD Deaths Due to High Intake of trans Fatty Acids	Percentage	Cumulative Percentage	Ranking by No. of CHD Deaths
United States	96,611	18.11	18.11	1
India	63,243	11.86	29.97	2
Egypt	44,257	8.3	38.27	3
China	42,356	7.94	46.21	4
Pakistan	40,239	7.54	53.75	5
Russia	29,480	5.53	59.28	6
Mexico	18,732	3.51	62.79	7
Brazil	18,576	3.48	66.27	8
Iran	14,398	2.70	68.97	9
Canada	13,901	2.61	71.58	10
Ukraine	12,980	2.43	74.01	11
Germany	10,916	2.05	76.06	12
Bangladesh	8,137	1.53	77.59	13
Indonesia	5,760	1.08	78.67	14
Japan	5,271	0.99	79.66	15
Poland	5,133	0.96	80.62	16
United Kingdom	4,712	0.88	81.50	17
Turkey	3,973	0.74	82.24	18
Belarus	3,561	0.67	82.91	19
Italy	3,381	0.63	83.54	20
France	3,084	0.58	84.12	21
Philippines	3,067	0.57	84.69	22
Netherlands	2,881	0.54	85.23	23
Romania	2,591	0.49	85.72	24
Uzbekistan	2,535	0.48	86.20	25
Thailand	2,517	0.47	86.67	26
Iraq	2,475	0.46	87.13	27
Colombia	2,215	0.42	87.55	28
Nepal	2,178	0.41	87.96	29
Sri Lanka	2,162	0.41	88.37	30

## Progress Toward Reduction of TFA Intake

**Figure 2:**
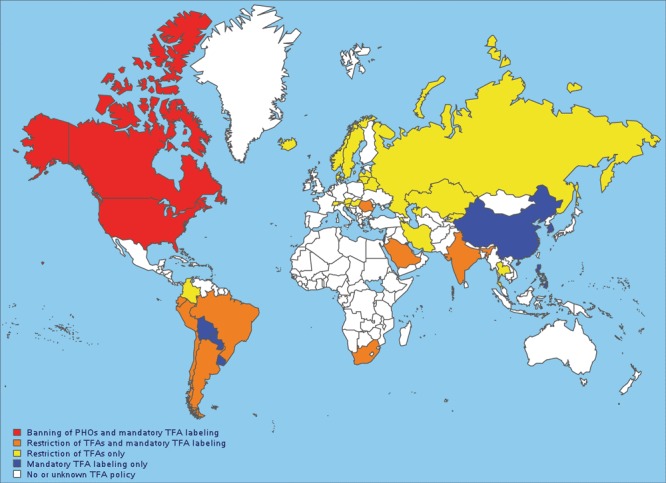
Countries with policies or regulations on industrially produced (artificial) TFAs. Data source: World Health Organization (14). Abbreviation: PHO, partially hydrogenated oil; TFA, *trans*-fatty acid.

The harmful health effects of TFAs, combined with high levels of iTFA intake, have motivated policy makers in many countries to take action. In the past 15 years, progress has been made toward reducing iTFA intake (10–13). In 2003, Denmark was the first country to enact a law to restrict the iTFA content of all food products and ready-to-eat meals, requiring that no more than 2% of the total fat content could come from iTFAs (10). Canada and the United States, where the daily dietary intakes of TFAs were more than twice the WHO-recommended limit of 1% of energy intake (5), were among the first countries to introduce mandatory labeling of TFAs in packaged foods in 2003; mandatory labeling went into effect in Canada in 2005 and the United States in 2006 (11,12). In the absence of national restrictions, numerous local jurisdictions in the United States, such as New York City, restricted TFAs in food service establishments, including restaurants, caterers, mobile food-vending units, and mobile food commissaries (13). In recent years, both the United States and Canada used existing food additive regulations to issue determinations that partially hydrogenated oils are not generally recognized as safe for any use in human foods, becoming the first countries to effectively ban iTFAs (11,12). To date, some 40 countries, most of which are high-income or upper-middle–income countries, have adopted mandatory restriction of iTFAs, banned the use of partially hydrogenated oils, and/or required mandatory labeling of TFAs on packaged foods (Figure 2).

**Table:** 

Country	Policy
Afghanistan	No or unknown TFA policy
Albania	No or unknown TFA policy
Algeria	No or unknown TFA policy
American Samoa	No or unknown TFA policy
Andorra	No or unknown TFA policy
Angola	No or unknown TFA policy
Anguilla	No or unknown TFA policy
Antarctica	No or unknown TFA policy
Antigua and Barbuda	No or unknown TFA policy
Argentina	Restriction of TFAs AND mandatory TFA labeling
Armenia	Restriction of TFA only
Aruba	No or unknown TFA policy
Australia	No or unknown TFA policy
Austria	Restriction of TFA only
Azerbaijan	No or unknown TFA policy
Bahamas	No or unknown TFA policy
Bahrain	Mandatory TFA labeling only
Bangladesh	No or unknown TFA policy
Barbados	No or unknown TFA policy
Belarus	Restriction of TFA only
Belgium	No or unknown TFA policy
Belize	No or unknown TFA policy
Benin	No or unknown TFA policy
Bermuda	No or unknown TFA policy
Bhutan	No or unknown TFA policy
Bolivia	Mandatory TFA labeling only
Bonaire, Saint Eustatius, and Saba	No or unknown TFA policy
Bosnia and Herzegovina	No or unknown TFA policy
Botswana	No or unknown TFA policy
Bouvet Island	No or unknown TFA policy
Brazil	Restriction of TFAs AND mandatory TFA labeling
British Indian Ocean Territory	No or unknown TFA policy
Brunei Darussalam	No or unknown TFA policy
Bulgaria	No or unknown TFA policy
Burkina Faso	No or unknown TFA policy
Burundi	No or unknown TFA policy
Cambodia	No or unknown TFA policy
Cameroon	No or unknown TFA policy
Cameroon/Nigeria	No or unknown TFA policy
Canada	Banning of PHOs AND mandatory TFA labeling
Cape Verde	No or unknown TFA policy
Cayman Islands	No or unknown TFA policy
Central African Republic	No or unknown TFA policy
Chad	No or unknown TFA policy
Chile	Restriction of TFAs AND mandatory TFA labeling
China	Mandatory TFA labeling only
China/India	No or unknown TFA policy
China/Taiwan, Province of China	No or unknown TFA policy
Christmas Island	No or unknown TFA policy
Cocos (Keeling) Islands	No or unknown TFA policy
Colombia	Restriction of TFA only
Comoros	No or unknown TFA policy
Congo	No or unknown TFA policy
Congo, Democratic Republic of the	No or unknown TFA policy
Cook Islands	No or unknown TFA policy
Costa Rica	No or unknown TFA policy
Cote D'Ivoire	No or unknown TFA policy
Croatia	No or unknown TFA policy
Cuba	No or unknown TFA policy
Curacao	No or unknown TFA policy
Cyprus	No or unknown TFA policy
Czech Republic	No or unknown TFA policy
Denmark	Restriction of TFA only
Djibouti	No or unknown TFA policy
Dominica	No or unknown TFA policy
Dominican Republic	No or unknown TFA policy
Ecuador	Restriction of TFAs AND mandatory TFA labeling
Egypt	No or unknown TFA policy
El Salvador	No or unknown TFA policy
Equatorial Guinea	No or unknown TFA policy
Eritrea	No or unknown TFA policy
Estonia	No or unknown TFA policy
Ethiopia	No or unknown TFA policy
Falkland Islands (Malvinas)	No or unknown TFA policy
Faroe Islands	No or unknown TFA policy
Fiji	No or unknown TFA policy
Finland	No or unknown TFA policy
France	No or unknown TFA policy
French Guiana	No or unknown TFA policy
French Polynesia	No or unknown TFA policy
French Southern Territories	No or unknown TFA policy
Gabon	No or unknown TFA policy
Gambia	No or unknown TFA policy
Georgia	No or unknown TFA policy
Germany	No or unknown TFA policy
Ghana	No or unknown TFA policy
Gibraltar	No or unknown TFA policy
Golan Heights	No or unknown TFA policy
Greece	No or unknown TFA policy
Greenland	No or unknown TFA policy
Grenada	No or unknown TFA policy
Guadeloupe	No or unknown TFA policy
Guam	No or unknown TFA policy
Guatemala	No or unknown TFA policy
Guernsey	No or unknown TFA policy
Guinea	No or unknown TFA policy
Guinea-Bissau	No or unknown TFA policy
Guyana	No or unknown TFA policy
Haiti	No or unknown TFA policy
Heard Island and McDonald Islands	No or unknown TFA policy
Holy See (Vatican City State)	No or unknown TFA policy
Honduras	No or unknown TFA policy
Hungary	Restriction of TFA only
Iceland	Restriction of TFA only
India	Restriction of TFAs AND mandatory TFA labeling
Indonesia	No or unknown TFA policy
Iran, Islamic Republic of	Restriction of TFA only
Iraq	No or unknown TFA policy
Ireland	No or unknown TFA policy
Isle of Man	No or unknown TFA policy
Israel	Mandatory TFA labeling only
Italy	No or unknown TFA policy
Jamaica	No or unknown TFA policy
Japan	No or unknown TFA policy
Jersey	No or unknown TFA policy
Jordan	No or unknown TFA policy
Kazakhstan	Restriction of TFA only
Kenya	No or unknown TFA policy
Kiribati	No or unknown TFA policy
Korea, Democratic People's Republic of	No or unknown TFA policy
Korea, Republic of	Mandatory TFA labeling only
Kosovo	No or unknown TFA policy
Kuwait	Mandatory TFA labeling only
Kyrgyzstan	Restriction of TFA only
Lao People's Democratic Republic	No or unknown TFA policy
Latvia	Restriction of TFA only
Lebanon	No or unknown TFA policy
Lesotho	No or unknown TFA policy
Liberia	No or unknown TFA policy
Libya	No or unknown TFA policy
Liechtenstein	No or unknown TFA policy
Lithuania	Restriction of TFA only
Luxembourg	No or unknown TFA policy
Macedonia, the Former Yugoslav Republic of	No or unknown TFA policy
Madagascar	No or unknown TFA policy
Malawi	No or unknown TFA policy
Malaysia	No or unknown TFA policy
Maldives	No or unknown TFA policy
Mali	No or unknown TFA policy
Malta	No or unknown TFA policy
Marshall Islands	No or unknown TFA policy
Martinique	No or unknown TFA policy
Mauritania	No or unknown TFA policy
Mauritius	Mandatory TFA labeling only
Mayotte	No or unknown TFA policy
Mexico	No or unknown TFA policy
Micronesia, Federated States of	No or unknown TFA policy
Moldova, Republic of	No or unknown TFA policy
Monaco	No or unknown TFA policy
Mongolia	No or unknown TFA policy
Montenegro	No or unknown TFA policy
Montserrat	No or unknown TFA policy
Morocco	No or unknown TFA policy
Mozambique	No or unknown TFA policy
Myanmar	No or unknown TFA policy
Namibia	No or unknown TFA policy
Nauru	No or unknown TFA policy
Nepal	No or unknown TFA policy
Netherlands	No or unknown TFA policy
New Caledonia	No or unknown TFA policy
New Zealand	No or unknown TFA policy
Nicaragua	No or unknown TFA policy
Niger	No or unknown TFA policy
Nigeria	No or unknown TFA policy
Niue	No or unknown TFA policy
Norfolk Island	No or unknown TFA policy
Northern Mariana Islands	No or unknown TFA policy
Norway	Restriction of TFA only
Oman	No or unknown TFA policy
Pakistan	No or unknown TFA policy
Palau	No or unknown TFA policy
Palestinian Territory, Occupied	No or unknown TFA policy
Panama	No or unknown TFA policy
Papua New Guinea	No or unknown TFA policy
Paraguay	Mandatory TFA labeling only
Peru	Restriction of TFAs AND mandatory TFA labeling
Philippines	Mandatory TFA labeling only
Pitcairn	No or unknown TFA policy
Poland	No or unknown TFA policy
Portugal	No or unknown TFA policy
Puerto Rico	No or unknown TFA policy
Qatar	No or unknown TFA policy
Reunion	No or unknown TFA policy
Romania	Restriction of TFAs AND mandatory TFA labeling
Russian Federation	Restriction of TFA only
Rwanda	No or unknown TFA policy
Saint Barthelemy	No or unknown TFA policy
Saint Helena	No or unknown TFA policy
Saint Kitts and Nevis	No or unknown TFA policy
Saint Lucia	No or unknown TFA policy
Saint Martin	No or unknown TFA policy
Saint Pierre and Miquelon	No or unknown TFA policy
Saint Vincent and the Grenadines	No or unknown TFA policy
Samoa	No or unknown TFA policy
San Marino	No or unknown TFA policy
Sao Tome and Principe	No or unknown TFA policy
Saudi Arabia	Restriction of TFAs AND mandatory TFA labeling
Senegal	No or unknown TFA policy
Serbia	No or unknown TFA policy
Seychelles	No or unknown TFA policy
Sierra Leone	No or unknown TFA policy
Singapore	Restriction of TFAs AND mandatory TFA labeling
Sint Maarten (Dutch Part)	No or unknown TFA policy
Slovakia	No or unknown TFA policy
Slovenia	Restriction of TFA only
Solomon Islands	No or unknown TFA policy
Somalia	No or unknown TFA policy
South Africa	Restriction of TFAs AND mandatory TFA labeling
South Georgia and the South Sandwich Islands	No or unknown TFA policy
Spain	No or unknown TFA policy
Sri Lanka	No or unknown TFA policy
Sudan	No or unknown TFA policy
Suriname	No or unknown TFA policy
Suriname/Guyana	No or unknown TFA policy
Svalbard and Jan Mayen	No or unknown TFA policy
Swaziland	No or unknown TFA policy
Sweden	Restriction of TFA only
Switzerland	Restriction of TFA only
Syrian Arab Republic	No or unknown TFA policy
Taiwan, Province of China	No or unknown TFA policy
Tajikistan	No or unknown TFA policy
Tanzania, United Republic of	No or unknown TFA policy
Thailand	Restriction of TFA only
Timor-Leste	No or unknown TFA policy
Togo	No or unknown TFA policy
Tokelau	No or unknown TFA policy
Tonga	No or unknown TFA policy
Trinidad and Tobago	No or unknown TFA policy
Tunisia	No or unknown TFA policy
Turkey	No or unknown TFA policy
Turkmenistan	No or unknown TFA policy
Turks and Caicos Islands	No or unknown TFA policy
Tuvalu	No or unknown TFA policy
Uganda	No or unknown TFA policy
Ukraine	No or unknown TFA policy
United Arab Emirates	No or unknown TFA policy
United Kingdom	No or unknown TFA policy
United States	Banning of PHOs AND mandatory TFA labeling
Uruguay	Mandatory TFA labeling only
Uzbekistan	No or unknown TFA policy
Vanuatu	No or unknown TFA policy
Venezuela	No or unknown TFA policy
Viet Nam	No or unknown TFA policy
Virgin Islands, British	No or unknown TFA policy
Virgin Islands, US	No or unknown TFA policy
Wallis and Futuna	No or unknown TFA policy
Western Sahara	No or unknown TFA policy
Yemen	No or unknown TFA policy
Zambia	No or unknown TFA policy
Zimbabwe	No or unknown TFA policy

Reductions in TFA intake, iTFAs in foods, and TFA-attributed CHD mortality have been reported in the United States, Denmark, and Argentina since the implementation of TFA policies (13,15–20). In the United States overall, from 1999–2000 to 2009–2010, plasma TFA levels decreased by 54% in adults aged 20 or older (15); both TFA food labeling and local regulations were enacted during this 10-year period. New York City’s 2007 regulation restricting TFAs in food-service establishments was associated with a significant reduction of 2.4 g in mean TFAs in fast-food purchases (13) and a greater decline in adult serum TFA levels among frequent restaurant diners (61.6%) than among people who rarely dined out (51.1%) (16). These declines show how the effect of the regulation in New York City added to the effect of labeling overall in the United States (16). Cardiovascular disease mortality was also affected, declining by 4.5% in New York City (17) and by 22 deaths per 100,000 person-year in Denmark (18). Restaurant restrictions in 11 counties in New York State were associated with an additional 6.2% decline, compared with 25 counties without TFA restrictions, in the hospital admission rate for myocardial infarction and stroke (19). Similarly, near elimination of iTFAs in Argentina was associated with a 1.3% to 6.3% reduction in CHD events in 2004 and 2014 (20).

Elimination of iTFAs from the food supply is politically viable, economically favorable, and technically feasible. Successful experiences in high-income countries such as the United States and Denmark can be transferred to LMICs. Some LMICs, such as Argentina, Brazil, Costa Rica, India, and Mexico, have taken steps to enact policies and/or create surveillance systems for monitoring TFA content in cooking oils and foods (21). However, most LMICs have no policies on iTFAs or have not enforced the policies they do have, despite the growing burden of CHD.

Making the case to restrict TFAs through regulation or legislation requires more than just the scientific case against TFAs. Information on the level of iTFAs in foods or on TFA intake are critical to motivate stakeholders and to evaluate the progress of eliminating partially hydrogenated oils in the food supply. To date, however, little is known about TFA intake in most LMICs (5), let alone the potential reduction in CHD burden and health benefits that could result from the elimination of iTFAs. For example, removal of partially hydrogenated oils in the United States was estimated to save $130 billion over 20 years and prevent 3,000 to 7,000 deaths due to CHD annually (12).

## Implications for Public Health Practice

### Need for objective and accurate measurement of TFA intake worldwide

In light of gaps in data on TFA intake, there is an urgent need to measure and monitor TFA intake globally. The current methods for estimating TFA intake are difficult to implement in LMICs. The primary method for estimating TFA intake is to collect data through food frequency dietary interviews in national health and nutrition surveys. In these surveys, the quantity of food products consumed is examined, and the TFA content is analyzed by consulting nutrition databases. Limitations of this method include incomplete or nonexistent information on TFA content in various food products and the reliance of dietary questionnaires on participant recall. Although it is possible to test popular foods for their TFA content, this testing is complex because TFA content may vary across brand (or vendor) and by geographic region. Furthermore, if countries are not already sampling and testing foods for other nutrients, new surveys would need to be developed and implemented. A second common method for estimating TFA intake is to survey a small sample of people and test duplicate portions of their meals for TFAs; this method is costly and complicated. A fast and effective approach for assessing the effect of policies is to measure TFA levels in blood. Such data are not currently available in many countries. The Centers for Disease Control and Prevention (CDC) pioneered a new technique for measuring TFAs in blood that was used on a subsample of the National Health and Nutrition Examination Survey in 1999–2000 and 2009–2010, allowing the assessment of change in TFA intake over time (15,22).

### Types of biological specimens for measuring TFAs

TFAs in the human body are derived from consumption of foods that contain TFAs; they cannot be synthesized by the human body. Therefore, TFA concentrations in human biologic tissues reflect dietary intake (2). Turnover rates or half-lives of fatty acids vary greatly from one part of the human body to another (2,3). Adipose tissue, with a slow turnover (average half-life of approximately 1 or 2 years), can be considered the best choice to assess long-term intake of TFAs. Red blood cells, with a moderate turnover (average half-life of approximately 1–4 months), can be used to assess medium-term intake. Plasma and serum, with a quick turnover (average half-life of approximately 5–10 days), are best used to assess short-term intake. Although adipose tissue might be ideal for assessing long-term intake, in practice, this approach is rarely used in large-scale epidemiologic studies and surveys because of the difficulty in obtaining samples. Specimens of plasma, serum, and red blood cells are widely used because of their accessibility; use of these specimens assumes relatively stable short-term dietary patterns. The standard laboratory protocol developed by CDC can be used to measure TFAs in plasma, serum, and red blood cells (erythrocytes) (22).

Whole blood has been proposed as an alternative specimen, and it could yield results on TFA intake that are similar to the results obtained by examining plasma (4). Moreover, the use of whole blood enables microsampling procedures (fingertip prick or heel prick) to collect dried blood-spot samples, which require low blood volume and simplified procedures for handling, storing, and processing samples. Indeed, dried blood-spot samples have been used to test many biomarkers (eg, hemoglobin, vitamin A, HIV) in the Demographic and Health Surveys Program in more than 50 countries (23). However, the dried blood-spot method is not widely used for fatty acid profiling because of its potential limitations (24). Oxidative degradation and filter paper contamination during sample storage and processing may compromise the accuracy of dried blood-spot assays for analyzing fatty acid composition. In addition, lack of consensus in analytic procedures or standard laboratory guidelines and difficulties in measuring fatty acid concentrations in the dried blood-spot samples may affect the reliability and comparability of laboratory data over time and across different populations.

### Practice of measuring TFAs in LMICs

Measuring TFAs as a part of national surveys in LMICs could be the most effective and direct way to understand TFA intake. Many LMICs collect blood samples (plasma or serum) as part of their national health or nutrition surveys, so it would be relatively simple to include TFA biomarkers as an additional test. The World Health Organization’s STEPwise Approach to Surveillance uses blood samples to test for cholesterol and blood glucose levels; this simple, standardized method for collecting, analyzing, and disseminating data has been implemented in more than 100 countries (25). National and regional laboratories would need to establish and build the technical and scientific capacity for measuring TFAs in blood and in foods. These laboratory measurements are complex and require a high-quality system to ensure accuracy and reliability of data over time and across countries and regions. High-quality systems would need to include common and standardized measurement protocols, training of personnel, and external quality assessment conducted within a network of laboratories. The data obtained from these laboratories would lead to the establishment of baseline levels of TFA intake, provide scientific evidence of the effect of public health policy making, and help with directing further actions to eliminate iTFAs in the food supply. With surveys and laboratories adhering to the same standards and using the same high-quality system, data can be compared with global references and across countries. Because laboratory measurements of TFAs also enable the measurement of other fatty acids, building laboratory capacity for measuring TFAs and other biomarkers for cardiovascular disease risk factors in blood enables countries and regions to address public health issues beyond iTFA reduction. Improving cardiovascular health through reduction of iTFA intake is feasible through shared experience, technical assistance, and advanced laboratory technology. It can be achieved by collaborating and partnering with key stakeholders in LMICs, and with public and private partners internationally.
